# Preclinical PET Imaging and Toxicity Study of a ^68^Ga-Functionalized Polymeric Cardiac Blood Pool Agent

**DOI:** 10.3390/pharmaceutics15030767

**Published:** 2023-02-25

**Authors:** Katayoun Saatchi, François Bénard, Navjit Hundal, Joshua Grimes, Sergey Shcherbinin, Maral Pourghiasian, Donald E. Brooks, Anna Celler, Urs O. Häfeli

**Affiliations:** 1 Faculty of Pharmaceutical Sciences, University of British Columbia, Vancouver, BC V6T 1Z3, Canada; 2 Department of Radiology, University of British Columbia, Vancouver, BC V5Z 1M9, Canada; 3 BC Cancer, Vancouver, BC V5Z 4E6, Canada; 4 Department of Pathology and Laboratory Medicine, University of British Columbia, Vancouver, BC V6T 1Z3, Canada

**Keywords:** Ga-68, hyperbranched polyglycerol, non-toxic polymer, macromolecule, blood pool imaging agent, PET radiopharmaceutical, internal dosimetry

## Abstract

Cardiac blood pool imaging is currently performed almost exclusively with ^99m^Tc-based compounds and SPECT/CT imaging. Using a generator-based PET radioisotope has a few advantages, including not needing nuclear reactors to produce it, obtaining better resolution in humans, and potentially reducing the radiation dose to the patient. When the shortlived radioisotope ^68^Ga is used, it can be applied repeatedly on the same day—for example, for the detection of bleeding. Our objective was to prepare and evaluate a long-circulating polymer functionalized with gallium for its biodistribution, toxicity, and dosimetric properties. A 500 kDa hyperbranched polyglycerol was conjugated to the chelator NOTA and radiolabeled rapidly at room temperature with ^68^Ga. It was then injected intravenously into a rat, and gated imaging allowed us to easily observe wall motion and cardiac contractility, confirming the suitability of this radiopharmaceutical for cardiac blood pool imaging. Internal radiation dose calculations showed that the radiation doses that patients would receive from the PET agent would be 2.5× lower than those from the ^99m^Tc agent. A complete 14-day toxicology study in rats concluded that there were no gross pathology findings, changes in body or organ weights, or histopathological events. This radioactive-metal-functionalized polymer might be a suitable non-toxic agent to advance for clinical application.

## 1. Introduction

While nuclear medicine blood pool agents are traditionally based on the ubiquitous gamma emitter ^99m^Tc with its favorable half-life, photon energy, and radiopharmaceutical chemistry [1,2], the use of PET radioisotopes in blood pool intravascular contrast agents might have a few potential advantages. First, quicker repeats between applications are possible due to the typical radioisotopes’ shorter half-lives, which would be advantageous for applications such as the detection of internal bleeding. Second, potentially lower radiation doses can be given [3,4]. Third, the resolution of the procedures will improve, as clinical PET imaging reaches a resolution of 4 to 6 mm, while SPECT imaging is typically between 8 and 12 mm [5]. Fourth, on-site production of PET radioisotopes is possible from both cyclotrons [6] and long-half-life radionuclide generators [7]. The non-availability of ^99m^Tc—for example, during the unplanned nuclear power plant shutdown of both the Petten reactor in the Netherlands and the NRU reactor in Canada in 2009—led to the loss of more than 50% of the global supply of this crucial radioisotope for several years, which was at that time used in more than 80% of all nuclear medicine procedures. A search for alternative methods of ^99m^Tc production [8] and alternative imaging agents [9,10,11,12] ensued.

Replacing some of the ^99m^Tc-based nuclear medicine diagnostic studies with positron emission tomography (PET)-based imaging procedures was thought to be one way of reducing global dependence on reactor-based radioisotopes. In this work, we investigated the use of a generator-produced PET isotope, ^68^Ga, for blood pool imaging. Previously, such studies were exclusively performed with single-photon emission computed tomography (SPECT) radioisotopes and allowed doctors to perform radionuclide angiography studies to investigate myocardial ejection fraction [13,14], diagnose gastrointestinal bleeding [15,16], and confirm cases of suspected hemangiomas [17].

^68^Ga has many appealing properties [18]. It is available from a ^68^Ge/^68^Ga generator that can be used for up to a year due to the long half-life (270.8 days) of its parent isotope ^68^Ge, which can be produced via cyclotron. Table 1 compares a list of the physical properties of ^68^Ga to other PET isotopes that have been investigated for blood pool imaging. Most of these PET isotopes are produced via cyclotron, and many have very short half-lives. Only ^82^Rb is currently used clinically for cardiac imaging [19]. Radiochemically, ^68^Ga is relatively easy to work with, although the best practices of ^68^Ga radiopharmacy should be followed, as conveniently outlined by Nelson et al. [20]. 

A blood pool agent is a non-toxic radiopharmaceutical that stays in circulation long enough to allow for easy nuclear imaging without distribution to other tissues. The most widely used clinically approved product for making such a blood pool imaging agent is the Ultratag^®^ kit. This allows users to very efficiently radiolabel red blood cells using three possible routes: one in vivo and two in vitro methods (Table 2). However, there are major drawbacks of using red blood cells as a blood pool imaging agent, as the in vitro approaches require handling of blood by the staff and inconvenience the patients due to multiple injections and waiting periods. Moreover, the easiest in vivo method, where red blood cells are radiolabeled inside a patient, unfortunately produces rather different red blood cell labeling efficiencies in diverse patient groups. For example, in cancer and transplant patients, their medication often interferes with the radiolabeling efficiency, and the diagnostic results can thus be suboptimal and even lead to misdiagnoses [22]. Having a universal kit to prepare the blood pool agent and eliminate these disadvantages would be ideal.

Several groups have worked on preparing a universal PET blood pool agent. For example, Wängler et al. radiolabeled rat serum albumin that was activated with sulfosuccinimidyl 4-(*N*-maleimidomethyl)cyclohexane-1-carboxylate (Sulfo-SMCC), with the PET isotope ^18^F in the form of 4-(di-tert-butyl[^18^F]fluorosilyl)benzenethiol, obtaining a radiochemical yield of 40–60% [23]. Basuli et al. also radiolabeled rat serum albumin with ^18^F with the help of a radiolabeled fluoronicotinic acid-2,3,5,6-tetrafluorophenyl ester, yielding a final radiochemical yield of 18–35% [24]. Another albumin-based blood pool agent was made by Wängler et al. with the PET isotope ^68^Ga [25]. Albumin was conjugated to Sulfo-SMCC, as in their previous paper, but then derivatized with the excellent gallium-chelating agent NODA-GA-T ((2,2′-(7-(1-carboxy-4-(2-mercaptoethylamino)-4-oxobutyl)-1,4,7-triazonane-1,4-diyl)diacetic acid). This chelator allows for kit-based radiolabeling at >95% efficiency with ^68^Ga without any further purification. Another group also radiolabeled albumin using a novel ^68^Ga-labeled maleimide-monoamide-DOTA, which rapidly bound albumin with a binding fraction above 70% [26]. They determined the biological half-life of this compound to be 190 min and successfully detected micro-bleeding in a rat model. Finally, Zhang et al. prepared an albumin-binding PET imaging probe that consisted of a ^68^Ga-radiolabeled dye (Evans blue) conjugated to ^68^Ga at >95% efficiency through the chelator NOTA (1,4,7-triazacyclononane-*N,N*′,*N*″-triacetic acid) [27]. Injected into the bloodstream, this dye binds to the most abundant serum protein albumin at high efficiency, forming an in vivo method similar to the in vivo Ultratag^®^ kit approach. The same NOTA-conjugated dye was successfully radiolabeled with another PET radioisotope (^64^Cu) by Niu et al. [28].

In our investigation, we aimed to prepare a kit-based PET imaging blood pool agent based on ^68^Ga, which was universally useful for all patients, would be radiolabeled within 10 min at room temperature outside the patient so that the radiolabeling does not interfere with their medications, and was not based on the blood product albumin. As a long-circulating polymer, we chose hyperbranched polyglycerol, which was developed by Haag et al. with molecular weights of up to 30 kDa [29], and then expanded in a one-pot synthesis by Kainthan et al. to molecular weights of up to 1 Mio kDa, still with excellent narrow size distributions [30]. These hyperbranched polyglycerols are abbreviated as HPG; they showed excellent biocompatibility, non-toxicity, and hemocompatibility [30,31], and they have a circulation half-life of between 10 and 48 h based on their molecular weight [32]. As the higher sensitivity and spatial resolution of clinical PET cameras compared to conventional nuclear imaging yields more accurate measurements of the ejection fraction, as well as more sensitive detection of gastrointestinal bleeding sites and small hemangiomas, we used dynamic PET imaging to investigate the potential of ^68^Ga-HPG as a blood pool imaging agent, and then we also performed a complete toxicology study that might establish it as a new investigational drug. The results are reported herein.

## 2. Materials and Methods

### 2.1. Materials

All chemicals were purchased from Sigma-Aldrich (St. Louis, MO, USA). The ^68^GaCl_3_ was received from MDS Nordion (Vancouver, BC, Canada). *p*-NH_2_-Bn-NOTA was purchased from Macrocyclics (Dallas, TX, USA). The ^1^H NMR spectra were recorded on a Bruker AV-300 or AV-400 at 300.13 or 400.13 MHz using deuterated solvents (99.8% D; Cambridge Isotope Laboratories, Andover, MA, USA), with the solvent peak as a reference. Sephadex™ G-25 M columns (PD10) were obtained from GE Healthcare (Piscataway, NJ, USA). Instant thin-layer chromatography (ITLC) was performed on green TEC-Control strips (Cat# 150-771; Biodex, Shirley, NY, USA). Dialysis tubing was obtained from Spectrum Laboratories (Rancho Dominguez, CA, USA), with a molecular weight cutoff (MWCO) of 1000. The radioactive TLCs were made visible by phosphor imaging (Cyclone storage phosphor imager with 20 × 25 cm^2^ phosphor screen, PerkinElmer, Waltham, MA, USA) and analyzed using OptiQuant software (PerkinElmer). Ultracel-YM100 microconcentrators were purchased from Millipore Corporation (Billerica, MA, USA).

### 2.2. Synthesis of NOTA-HPG and Radiolabeling

The high-molecular-weight polyglycerol (HPG) was synthesized in Brooks’ lab according to published procedures [33], and then derivatized with *p*-NH_2_-Bn-NOTA to yield NOTA-HPG [34]. ^1^H NMR (D_2_O, 300 MHz): δ 2.5–4.0 (*b*, overlapping multiplets, HPG [-C*H*_2_-C*H*C*H*_2_-OH-O]*_n_*, NOTA N-*CH_2_CH_2_*-N and N-*CH_2_*-COOH); 7.2–7.6 (d, NOTA CH_2_-*C_6_H_5_*). GPC = *M_n_* 600 kDa; PDI = 1.101 ± 0.007; number of NOTA chelators per HPG = 52, calculated by ICP analysis after coordination with non-radioactive gallium. The polymer was then radiolabeled by adding the radioactive ^68^GaCl_3_ (7.4–370 MBq) in HCl (0.1 N) to a freeze-dried kit made from NOTA-HPG (2 mg, 3 nmols) in NH_4_OAc (500 μL, 0.6 M) and dextrose (5%, 500 µL). After 10 min at room temperature, the pH was adjusted to neutral with NaOH (1 N), and instant thin-layer chromatography (ITLC) was performed with 100 μL of a 33.3% mixture of saline, 0.1 M HCl, and 0.1 M Na_2_EDTA as the mobile phase. In this ITLC system, ^68^Ga-EDTA moves to R_f_ = 1, while ^68^Ga-HPG stays at R_f_ = 0. ITLC was also performed on the original ^68^GaCl_3_ solution and developed using a phosphor imager. The stability of the ^68^Ga-chelator-HPG construct was also measured over 2 h at 37 °C with an EDTA and transferrin challenge, as previously described by our lab [34]. After incubation, HPG-bound ^68^Ga and free radioactivity were separated at 1 and 2 h on a PD10 size-exclusion column.

### 2.3. Cardiac Blood Pool Imaging

The cardiac blood pool imaging properties of ^68^Ga-HPG were determined in a group of 5 female Sprague Dawley rats weighing 225 ± 15 g, in accordance with protocols approved by the University of British Columbia Animal Care Committee and the Canadian Council of Animal Care guidelines. The animals received a tail vein injection of the radiopharmaceutical (2 mg in 0.5 mL of PBS, 20 MBq) while placed on the scanner bed. All images were acquired on a Siemens Inveon animal PET/CT scanner at the British Columbia Cancer Agency (Vancouver, BC, Canada). First, a whole-body CT scan was performed for attenuation correction. A dynamic list-mode dataset was then acquired over 30 min to capture the first-pass and equilibrium imaging with ^68^Ga-HPG. Cardiac gating was performed with the Biovet (m2m Imaging Inc., Cleveland, OH, USA) system, supplied by the scanner. To assess the stability and confirm the biodistribution of the ^68^Ga-HPG by imaging, additional images were acquired at 1–1.5 and 2–2.5 h post-injection. The rats were kept warm by using the integrated Biovet bed warmer during acquisition, under isoflurane sedation. Between imaging sessions, the animals were awake in a cage. After the experiment, the animals were euthanatized by CO_2_ inhalation while under isoflurane anesthesia.

To investigate the nature of the radioactivity excreted through the kidneys, a combined urine sample from two rats collected during the first hour after injection was separated by size-exclusion chromatography on a Sephadex G25 column (PD10™, GE Healthcare Bio-Sciences Corp., Piscataway, NJ, USA) and analyzed by gamma counting.

### 2.4. Image Reconstruction and Analysis

The data were reconstructed using the Inveon Acquisition Workplace (IAW) software provided by Siemens. The list-mode acquisition allowed us to retrospectively rebin the sinograms for dynamic imaging with the time intervals of our choice. We chose 10 s intervals for the first 5 min to visualize the first pass of the tracer in the heart chambers, followed by a 20 min gated reconstruction (10-30 min) to evaluate wall motion and ejection fraction. Gated datasets were binned into 8 gates per cardiac cycle and reconstructed using an iterative algorithm (OSEM-3D/MAP), yielding a total of 8 three-dimensional images depicting ^68^Ga-HPG distribution in the heart cavities. Static images from the 1 and 2 h acquisitions were reconstructed using the OSEM-3D/MAP algorithm.

The images were then imported into the Inveon Research Workplace (IRW) data analysis program from Siemens. Regions of interest (ROIs) were drawn around organs, and the software calculated the percentage of injected activity per gram (%IA/g) in each region and the volume of each region using the actual injection volume, activity, and rat weight. For static 1 and 2 h images, the ROIs were drawn around the heart, liver, bladder, growth plate, bone, muscle, spine, and the right and left kidneys. For the 30 min dynamic images, ROIs were drawn around the same organs and tissues for the data collected at 5, 10, 15, 20, 25, and 30 min.

### 2.5. Gating and Ejection Fraction Calculations

This analysis was performed using the IRW workstation, using the 20 min gated images. Three-dimensional ROIs were drawn manually, closely outlining the left ventricle in each bin for the LVEF, and outlining the right ventricle in each bin for the RVEF. The volume of the regions and %IA/g of the regions were calculated by the program for each bin. The ejection fractions were calculated using the images by taking the counts from the ROIs made on the end-diastolic (ED) phase and the end-systolic (ES) phase. Since the counts are proportional to the volume, the ejection fraction could be determined by subtracting ES from ED volumes and dividing by the ED volume.

### 2.6. Radiation Dosimetry

The %IA/g values in the heart, liver, muscle, and kidneys in each of the 5 rats were converted to the %IA in the corresponding human organs of a male human reference phantom [35], using the following formula:

$${\left(\frac{\%\mathrm{IA}}{g}\right)}_{\mathrm{rat}\;\mathrm{organ}}\times \mathrm{rat}\;\mathrm{weight}\;\left(\mathrm{kg}\right)\times {\left(\frac{\mathrm{organ}\;\mathrm{weight}\;\left(g\right)}{\mathrm{total}\;\mathrm{body}\;\mathrm{weight}\;\left(\mathrm{kg}\right)}\right)}_{\mathrm{human}}={\left(\frac{\%\mathrm{IA}}{\mathrm{organ}}\right)}_{\mathrm{human}}$$

The calculated human %IA/organ data were reverse decay corrected and plotted versus time. The time–activity data were integrated using the trapezoidal method [36] to determine the time-integrated activity coefficient (TIAC) for each organ. To estimate the total number of decays after the last data point, elimination of radioactivity was assumed to be due to physical decay alone.

A conservative estimate of the total number of disintegrations occurring in the body was made by assuming no excretion, in which case the TIAC for the total body is 1/λ_p_ (where λ_p_ represents the physical decay constant for ^68^Ga) and is equal to 0.615 h^−1^. Finally, the TIAC for the remainder of the body was determined by subtracting the calculated TIACs for the heart, liver, muscle, and kidneys from the TIAC of the total body.

The TIACs for the heart, liver, muscle, kidneys, and remainder of the body were used as inputs for radiation dose calculation with the OLINDA/EXM 1.1 software, using the adult male reference phantom [35].

### 2.7. Comprehensive Toxicology Studies

A total of 40 Sprague Dawley rats were arranged into groups of 10/sex/group and received either 200 µL of Ga-HPG or 5% dextrose injection USP (D5W) as a single intravenous bolus injection. On day 1, the rats were between 7.5 and 9.5 weeks of age; males weighed 217–247 g, and females weighed 188–216 g. The test dose of 0.86 mg Ga-HPG in 200 µL of D5W was approximately 150× the dose anticipated to be administered in human subjects. Rats were monitored for 14 days after pharmaceutical administration for mortalities, clinical signs, and the times of onset, duration, and reversibility of toxicity. Gross necropsies were performed on all animals. All animals were monitored for 14 days following pharmaceutical administration and euthanatized on study day 15, as described above.

#### 2.7.1. Hematology

Blood was drawn from the animals and shipped to the analytical laboratory (IDEXX Laboratories; Vancouver, BC, Canada) on ice by same-day delivery and same-day processing for complete blood counts with differentials. Hematological assays included a complete blood count (i.e., white cell count, red blood cells, hemoglobin, hematocrit, mean corpuscular volume, mean corpuscular hemoglobin, mean corpuscular hemoglobin concentration, red cell distribution width, platelets, mean platelet volume) and differential test (i.e., neutrophils, lymphocytes, monocytes, eosinophils, and basophils).

#### 2.7.2. Blood Chemistry

Blood was drawn from the animals and processed to serum. Serum samples were shipped to the analytical laboratory (IDEXX Laboratories) on ice by same-day delivery and same-day processing for blood chemistry analysis. Blood chemistry evaluation included the following tests: glucose, urea, creatinine, blood urea nitrogen/creatinine ratio, sodium, potassium, sodium/potassium ratio, chloride, bicarbonate, anion gap, calcium, phosphorus, total protein, albumin, globulin, albumin/globulin ratio, total bilirubin, alkaline phosphatase (ALP), alanine transaminase (ALT), aspartate aminotransferase (AST), gamma-glutamyl transpeptidase (GGT), creatinine kinase (CK), and calculated osmolality.

#### 2.7.3. Necropsy/Gross Pathology

A necropsy was performed on each animal and the gross pathological findings were recorded on study day 15. The organs/tissues examined during the necropsy included the following: all gross lesions, as well as the brain (i.e., cerebral cortex, midbrain, cerebellum, and medulla), pituitary gland, eyes, thymus, thyroid and parathyroid, tongue, esophagus, salivary gland, stomach, small and large intestines, colon, liver, kidneys, adrenals, pancreas, spleen, heart, trachea, lungs, aorta, gonads, uterus, accessory sex organs, mammary glands, prostate (males), urinary bladder, lymph nodes, skeletal muscle, and skin. The following organs were also weighed at the time of necropsy: liver, spleen, kidneys, lung, heart, and brain 

#### 2.7.4. Histopathology

After a necropsy and gross pathology examination on each animal, selected organs and tissues were removed, fixed, and embedded in paraffin. Sectioning and H&E staining were performed on 6 of the 10 male and female Sprague Dawley rats by random selection and submitted for histopathological evaluation. All tissue embedding in paraffin, sectioning, and H&E staining were performed according to standard procedures implemented by Wax-it Histology Services (Vancouver, BC, Canada).

#### 2.7.5. Statistical Methods

The data in this study were subjected to calculation of group means and standard deviations. Significance was determined by the two-tailed *t*-test.

## 3. Results

**Figure 1:**
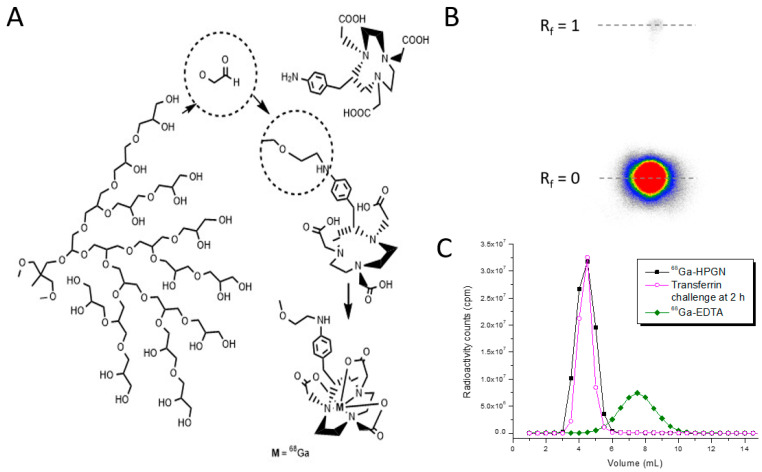
(**A**) Schematic synthesis of ^68^Ga-HPG. (**B**) Radiolabeling efficiency measured by thin-layer chromatography was above 99%. In the mobile phase mixture (33.3% each of saline, 0.1 M HCl, and 0.1 M Na_2_EDTA), the free ^68^Ga chelated by EDTA moved to the front, while the radiolabeled ^68^Ga-HPG stayed at the origin. (**C**) Size-exclusion chromatography (GPC) was used to measure the stability of ^68^Ga-HPGN. Only a single peak (black) was measured. A challenge with transferrin at 100× excess proved the very high stability of the product—no ^68^Ga was transchelated to the position where ^68^Ga-transferrin or ^68^Ga-EDTA was found at ~8 mL.

NOTA-HPG was labeled at room temperature in just 10 min, with a labeling efficiency of 98.7 ± 0.5% (see the thin-layer chromatogram in Figure 1), and then directly used for imaging. The ^68^Ga was very stably bound to HPG, as 99.3% and 98.9% was still polymer-bound after 1 h and 2 h of incubation in 0.1 M EDTA solution, respectively. Measured against a 100× excess of transferrin, the stability was even higher, at >99% at both the 1 h and 2 h time points. The stability of the radiopharmaceutical is thus expected to be better than 99% during the entire time of it being radioactive.

**Figure 2:**
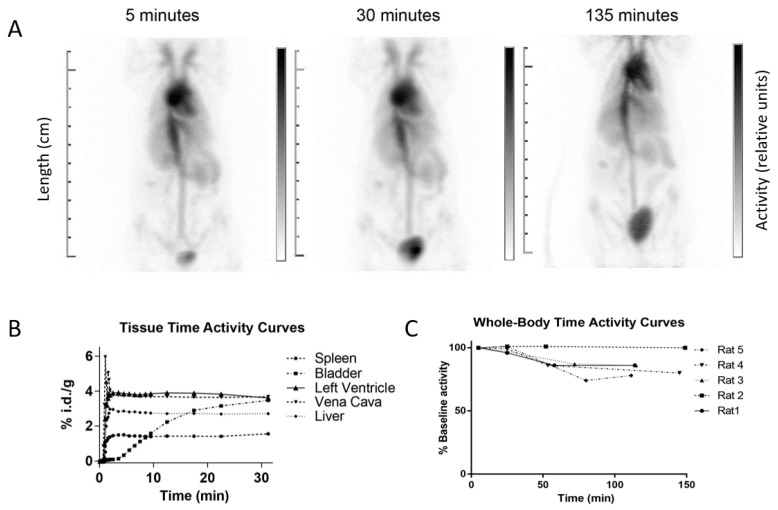
(**A**) ^68^Ga-NOTA-HPG biodistribution measured over time by PET imaging shows the typical blood pool imaging pattern. (**B**) Mean ± SD time–activity curves of ^68^Ga-HPG from the dynamic acquisition in different tissues. (**C**) The whole-body time–activity curves of all animals are shown over 2.5 h (*n* = 5).

The ^68^Ga-HPG biodistribution assessed visually followed a typical blood pool imaging pattern, with activity present in the heart cavities, main blood vessels and, to a lesser extent, in richly vascularized organs such as the spleen. Some urinary bladder excretion is visible on delayed images (Figure 2A). Typical time–activity curves from the dynamic acquisition are shown in Figure 2B. Overall, no activity was present in the gut, and most of the radioactivity remained confined in the blood vessels. A representative movie of the gated blood pool imaging is available as Supplementary Video S1.

**Figure 3:**
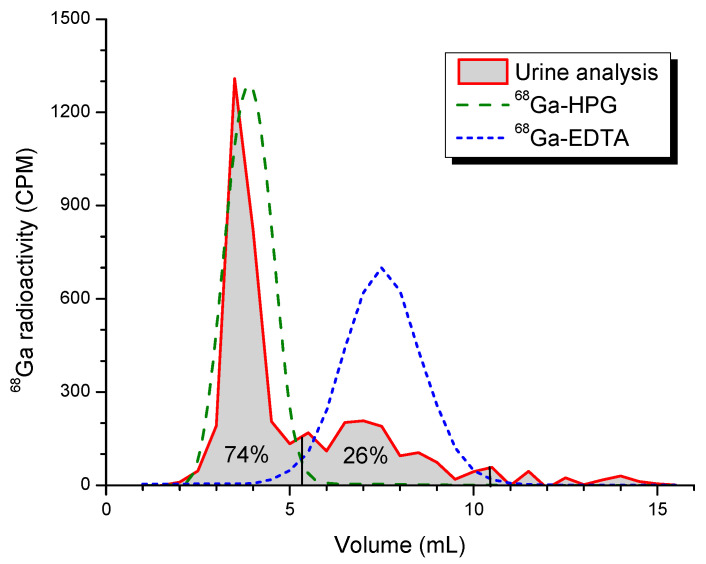
Size-exclusion chromatography of rat urine collected during the first hour after injection of ^68^Ga-HPG into a rat. The main peak of the urine activity (74%) came at the front, where pure ^68^Ga-HPG (green peak) was eluted. The other 26% of the urine activity was eluted where chelated ^68^Ga elutes (the blue peak), and might thus consist of partly broken down low-molecular-weight ^68^Ga-HPGs or other chelated ^68^Ga species.

After 2 h, on average 86.2 ± 8.7% of the initial activity remained in the rat, indicating that 14% of the radioactivity was excreted in the urine at that time. The whole-body time–activity curves for the individual rats are shown in Figure 2C. While the activity diminished similarly over time in four of the rats, rat 2 had not yet urinated by the last time point, as seen from the bladder activity at the final time point (not shown), and this is why its whole-body activity stayed constant. The analysis of a urine sample by size-exclusion chromatography (Figure 3) showed that more than 70% of the radioactive species in the urine consisted of a highly water-soluble macromolecular compound, and not of free ^68^Ga or ^68^Ga-NOTA complexes.

**Figure 4:**
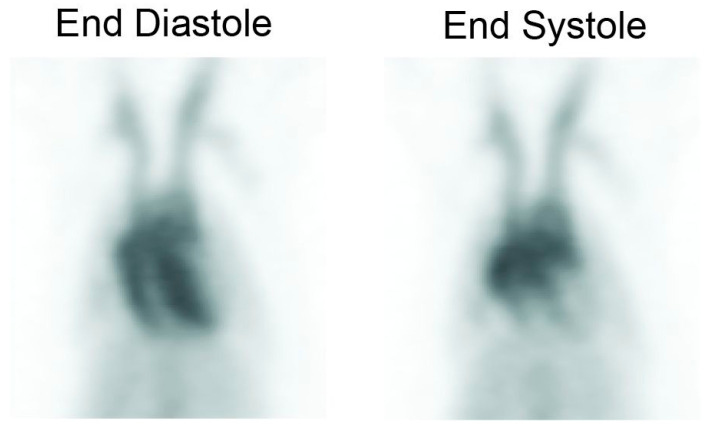
Gated blood pool images of a rat, rebinned using 8 gates per frame. The left image is from gate 8 (end diastole), while the right image shows gate 3 (end systole).

The ejection fraction data were calculated from individual gated datasets (e.g., Figure 4). The average ejection fraction was 68.9 ± 9.8%. This is consistent with the results obtained by other investigators using gated SPECT acquisitions performed with a pinhole collimator (^99m^Tc sestamibi and ^99m^Tc red blood cells) [37], where values of 72–74% and 63–65% were obtained, respectively. These values are slightly lower than published values using ^18^F-FDG PET and ultrasound in rats [38]. In mice, other investigators reported ejection fractions of 68 ± 6% using PET and 66 ± 4% using MRI [39].

The right ventricular ejection fractions of the rats were 46.94 ± 2.93%, which is lower than the 66 ± 2% reported in healthy Sprague Dawley rats [40]. The differences might have to do with the different imaging techniques (CT-attenuated SPECT vs. portable gamma camera) or the type of anesthesia (isoflurane vs. pentobarbital), which can influence the EF via the stroke volume [41,42].

Radiation dose estimates resulting from a 148 MBq injection of ^68^Ga, averaged over the five subjects, are summarized in Table 3. The effective dose estimated for a human subject is 1.8 mSv for a 148 MBq dose of ^68^Ga-HPG, compared to an effective dose of 4.5 mSv for the most commonly used dose (740 MBq) of ^99m^Tc-labeled RBCs, for all of the different in vitro*/*in vivo doses mentioned in Table 2 [43,44]. The Society of Nuclear Medicine recommends a dose between 555 MBq and 1110 MBq for ^99m^Tc-labeled RBCs [45], which means that the range of effective doses delivered with the currently used procedures would span from 3.4 to 6.8 mSv.

While the radiation doses from the ^68^Ga-HPG blood pool agent are between 1.9× and 3.8× lower than those of ^99m^Tc-labeled RBCs, the dose from the CT typically performed with PET imaging will add to the procedure. In clinical imaging, CT typically adds 2–20 mSv for the attenuation scan [46]. The rather large range in this dose comes from the type of PET/CT scanner and the chosen field for the CT. However, new ultralow-dose CT attenuation correction scans reduce these doses significantly, to 0.4 to 1.6 mSv [47,48], and Partington et al. reported even a lower attenuation correction CT dose of 0.3 mSv [3].

Ga-HPG, at 150 times the amount expected to be used clinically, was well tolerated for 14 days following administration in both male and female rats (group size of *n* = 10). All animals survived to the scheduled end date. The health scores were most often “0”, indicating no observations during the study; in rare cases, the rats had porphyrin staining on their nose, neck, and/or eyes. However, this is a common observation in naïve rats, was observed in rats receiving only 5% dextrose as well, and was not attributed to the administration of Ga-HPG.

**Figure 5:**
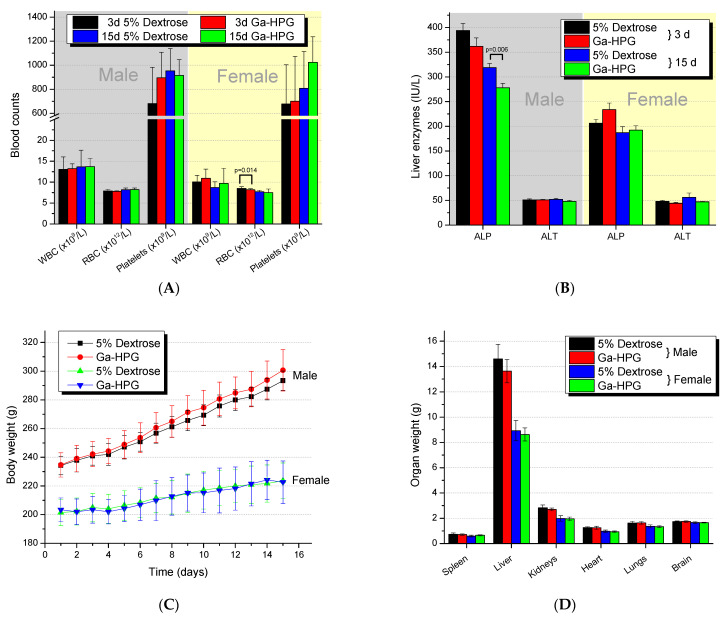
(**A**) Blood counts and (**B**) liver enzyme levels in male and female Sprague Dawley rats 3 and 15 days after application of Ga-HPG at 150x the amount given during the imaging (*n* = 10 per group and sex). (**C**) Body weight over time. (**D**) Organ weight at time of euthanasia (day 15) in male and female Sprague Dawley rats after application of Ga-HPG at 150× the amount given during the imaging (*n* = 10 per group and sex).

The only statistically significant difference in complete blood counts (Figure 5A) in male or female Sprague Dawley rats receiving Ga-HPG compared to 5% dextrose solution was for the RBC counts of females (day 3; *p* = 0.014). However, on day 15, the numbers were within normal counts of 8.6 ± 0.4 × 10^12^ RBCs/L for females. The only statistically significant difference for blood chemistry analysis (Figure 5B) was observed for the liver enzyme ALP in males on day 15 (*p* = 0.006), which was 13% lower than the ALP of the male animals’ control group. More detailed information is given in Supplementary Figures S1–S3.

The histopathology report concluded that there were no gross pathology findings, as well as no changes in mean group body weights, body weight gains (Figure 5C), organ weights (Figure 5D), organ-to-body weight ratios, organ-to-brain weight ratios, or histopathological findings attributed to Ga-HPG.

## 4. Discussion

Blood pool imaging is a common diagnostic procedure that is used to assess left ventricular function, as well as for the detection of liver hemangiomas and gastrointestinal bleeding. Most commonly, the tools used for these nuclear medicine diagnoses are radiolabeled red blood cells [13,14]. Blood pool imaging has been used successfully for over 40 years to assess ventricular dysfunction—first with ^99m^Tc-labeled albumin, and then almost exclusively with ^99m^Tc-labeled red blood cells (RBCs)—due to lower liver uptake and higher target-to-background ratios [49]. Despite advances in alternative imaging modalities such as ultrasound and magnetic resonance imaging, blood pool imaging with radiolabeled RBCs remains an important routine clinical tool. However, due to long-term sustainability issues with the reactor supply of ^99m^Tc’s precursor ^99^Mo, new ^99m^Tc production has been investigated and is now coming online. Replacing the procedure with PET is another option, and here we investigated blood pool imaging with the generator PET isotope ^68^Ga.

For a radiopharmaceutical to be considered useful in the clinic, it must be highly stable in vivo and easy to prepare. Radiolabeling of HPG with ^68^Ga carried out at room temperature was fast and showed excellent labeling efficiency, always exceeding 97%. The good stability of ^68^Ga-HPG for in vivo use was predicted by challenging the bound Ga with the competitive ligands EDTA and apotransferrin. Transferrin, the iron transporter protein, easily scavenges Ga^+3^, as it has the same size and charge as Fe^+3^ (0.620 vs. 0.645 Å). Both EDTA and transferrin bind Ga^+3^ with similar stability [50]. However, since NOTA has a much stronger binding affinity to Ga^+3^, no transchelation was expected nor observed in vitro. In vivo, we noted some urinary excretion of ^68^Ga, which accounted for an approximately 14% elimination on average, after 2 h. This is similar to what we observed previously in rats receiving ^67^Ga-HPG [34]. The mechanism for ^68^Ga excretion is unknown and needs further investigation.

Other radiotracers have been reported but are not in common clinical use for blood pool imaging by positron emission tomography. For example, methods have been reported to label human serum albumin with ^68^Ga [51], and carboxyhemoglobin can be labeled with ^11^CO or C^15^O (carbon monoxide) [52,53].

Our approach has the benefit of a simple kit formulation that avoids the use of biological materials of human origin. Furthermore, there is no need for an on-site cyclotron, and the activity can be obtained conveniently from an on-site, FDA-approved ^68^Ge/^68^Ga generator that is useful for up to 1 year and is currently available from Cardinal Health’s IRE ELiT Galli Eo^®^ [54] and ITM Medical Isotopes/RadioMedix GeGant^®^ [55] in clinical quality.

Despite the higher potential cost of ^68^Ga and overall higher costs of PET compared to conventional nuclear imaging, ^68^Ga-HPG imaging has several potential advantages over ^99m^Tc-labeled RBCs for blood pool imaging. The higher sensitivity and spatial resolution of PET imaging could potentially allow the user to resolve small hemangiomas with higher accuracy and perform more accurate blood volume estimates for ejection fraction calculation. Factors that affect red blood cell labeling—such as chemotherapy drugs and heparin—would not degrade image quality. The lack of gastric excretion would also make ^68^Ga-HPG an ideal candidate for the sensitive detection of gastrointestinal bleeding. Finally, this tracer avoids the need for blood handling by technologists, while keeping the ease of use of the kit formulation for rapid routine labeling, which can be performed multiple times daily with a ^68^Ge/^68^Ga generator. On the patient side, the waiting times currently needed between the potentially multiple injections necessary for optimal results (Table 2) would not be necessary, making the procedure much simpler and more convenient for the patients, as well as for the nurses. Furthermore, the doctors would not have to choose between the different methods, and imaging results would be expected to always be optimal.

HPG-Ga at much higher doses (150× higher) than expected to be used in vivo was well tolerated following intravenous injection in both male and female animals, with no body weight loss observed for the duration of the study. There were no clinical observations attributed to the Ga-HPG in terms of complete blood counts/differentials or blood chemistry analyses, gross pathology findings, or histopathology findings. There were also no significant changes observed between male and female Sprague Dawley rats.

## 5. Conclusions

To overcome the many issues of the currently used clinical practice for cardiac blood pool imaging, we developed a non-toxic polymeric macromolecule that is easily labeled by ^68^Ga using a simple kit formulation. The simplicity of using ^68^Ga-HPG makes it a strong candidate as a universal PET blood pool imaging agent.

Such a PET blood pool imaging agent would be of value not only where the ^99m^Tc blood pool agent is not available due to radioisotope shortage, but also where a higher clinical resolution is needed and where radiation doses are of concern and need to be minimized.

Another potential application of the NOTA-containing biocompatible polymer is in the theranostic area for the delivery and accumulation of both diagnostic and therapeutic radioisotopes into tissues with leaky vessels, such as are present in actively growing tumors. The present enhanced permeation and retention effect (EPR) leads to higher concentrations of macromolecules. The chemically conjugated NOTA chelator on HPG also strongly binds the therapeutic alpha emitters ^213^Bi and ^225^Ac, as well as the beta emitters ^177^Lu and ^155^Tb, and can then internally irradiate the tumor tissue [56]. However, more work needs to be carried out in optimizing the molecular weight of HPG and maximizing its uptake into tumors [32].

## Figures and Tables

**Table 1:** PET tracers investigated for blood pool imaging (taken from Conti and Eriksson [21]).

Tracer	T_1/2_ (min)	β^+^ (%)	β^+^ Max. Energy (keV)	β^+^ Mean Energy (keV)	β^+^ Max Range (mm)	β^+^ Mean Range (mm)	Production Routes
^13^N	9.97	99.8	1198.5	491.8	5.5	1.2	^16^O(p,α)
^15^O	2.03	99.9	1732.0	735.3	8.4	3.0	^15^N(p,n)
^18^F	110	97	633.5	249.8	2.4	0.6	^18^O(p,n)
^38^K	7.6	99.3	2724.4 *	1211.4	12.1	5.5	^40^Ar(p,3n)
^82^Rb	1.27	95.2	3378	1479	17.0	7.1	^82^Sr/^82^Rb generator
^94m^Tc	52	72	2439	1072	11.1	4.8	^94^Mo(p,n)
^68^Ga	68	89	1899.1	836.0	9.2	3.5	^68^Ge/^68^Ga generator

* The $\beta^{+}$ maximum energy for this radioisotope is actually 4891.9 keV, but very weak (0.05%). The dominant $\beta^{+}$ branch that is listed here has an intensity of 99.3% and a corresponding maximum range of 12.1 mm.

**Table 2:** Currently used clinical techniques for radiolabeling RBCs, compared to the method presented here.

Current Methods				New Method
In Vivo	Modified In Vivo/In Vitro	In Vitro 1	In Vitro 2	Long-Circulating Polymer
Reconstitute Ultratag^®^ kit with saline	Reconstitute Ultratag^®^ kit with saline	Withdraw 3 mL of blood and place in empty vial	Withdraw 3 mL of blood	Add ^68^Ga solution to kit
Inject measured dose into patient’s vein	Inject measured dose into patient’s vein	Add freshly made tin and EDTA solution and saline	Add blood to Ultratag^®^ kit	Incubate at RT for 2–5 min
Wait 20–30 min	Wait 20 min	Centrifuge	Incubate for 5 min	Perform quality control, ~10 min
Inject ^99m^Tc pertechnetate solution into patient	Place i.v. catheter into patient and draw 5 mL blood	Wait 2 min	Add contents of syringes 1 and 2 from the kit to the vial; mix by inversion	If labeling > 95%, inject into patient within the next 1 h
Image	Add ^99m^Tc pertechnetate through stopcock and cover with syringe shield	Collect RBCs—not plasma—and add to vial with ^99m^Tc pertechnetate	Place in lead shield and add ^99m^Tc pertechnetate, mix	Image
	Wait 5 min	Wait 5 min	Wait 20 min	
	Reinject into patient	Perform quality control, ~10 min	Perform quality control, ~10 min	
	Image	If labeling > 95%, inject into patient within the next 2 h	If labeling > 95%, inject into patient within 30 min	
		Image	Image	
≪80% *	90% *	>95% *	>95% *	>95%

* Typical labeling efficiencies. In chemotherapy patients, labeling efficiencies with the in vivo method are often around 50%.

**Table 3:** Comparison of the radiation dosimetry of the new blood pool agent ^68^Ga-HPG and conventional ^99m^Tc-labeled RBCs (from Stabin et al. [43]), using the average * recommended imaging dose.

	^68^Ga-HPG (mSv/148 MBq)				^99m^Tc-RBCs (mSv/740 MBq *)
Target Organ	Average	S.D.	Min	Max	
Adrenals	1.9	0.1	1.7	2.0	4.0
Brain	1.5	0.2	1.2	1.7	2.0
Breasts	1.4	0.1	1.3	1.6	2.4
Gallbladder wall	2.0	0.1	1.9	2.2	3.8
LLI wall	1.7	0.1	1.5	1.8	3.4
Small intestine	1.8	0.2	1.6	1.9	3.3
Stomach wall	1.7	0.1	1.5	1.9	3.4
ULI wall	1.8	0.2	1.6	1.9	3.3
Heart wall	8.0	0.5	7.3	8.5	11.8
Kidneys	6.3	0.6	5.8	7.1	5.1
Liver	6.1	1.0	4.8	7.1	4.8
Lungs	1.7	0.1	1.5	1.8	8.9
Muscle	1.4	0.3	1.1	1.8	2.7
Ovaries	1.7	0.1	1.5	1.9	3.5
Pancreas	1.9	0.1	1.7	2.0	4.2
Red marrow	1.3	0.1	1.2	1.5	3.0
Osteogenic cells	2.1	0.2	1.9	2.4	4.9
Skin	1.3	0.1	1.1	1.4	1.7
Spleen	1.7	0.1	1.5	1.8	8.9
Testes	1.4	0.1	1.3	1.6	2.4
Thymus	1.7	0.1	1.6	1.9	4.2
Thyroid	1.5	0.1	1.3	1.7	2.7
Urinary bladder wall	1.6	0.1	1.5	1.8	11.1
Uterus	1.7	0.1	1.5	1.9	4.0
**Effective Dose**	**1.8**	0.1	1.6	1.9	**4.5**

## Data Availability

The data presented in this study are available on request from the corresponding author.

## Supplementary Materials

The following supporting information can be downloaded at: https://www.mdpi.com/article/10.3390/pharmaceutics15030767/s1, Video S1: A movie of the beating heart reconstructed from the gated 68Ga-HPG PET images. Figures S1 to S3: Additional complete blood counts/differentials and blood chemistry analyses from the toxicology study.

## References

[1] Bartholoma M.D., Louie A.S., Valliant J.F., Zubieta J. (2010). Technetium and gallium derived radiopharmaceuticals: Comparing and contrasting the chemistry of two important radiometals for the molecular imaging era. Chem. Rev..

[2] Dilworth J.R., Parrott S.J. (1998). The biomedical chemistry of technetium and rhenium. Chem. Soc. Rev..

[3] Partington S.L., Valente A.M., Bruyere J., Rosica D., Shafer K.M., Landzberg M.J., Taqueti V.R., Blankstein R., Skali H., Kwatra N. (2021). Reducing radiation dose from myocardial perfusion imaging in subjects with complex congenital heart disease. J. Nucl. Cardiol..

[4] Al Badarin F.J., Spertus J.A., Bateman T.M., Patel K.K., Burgett E.V., Kennedy K.F., Thompson R.C. (2020). Drivers of radiation dose reduction with myocardial perfusion imaging: A large health system experience. J. Nucl. Cardiol..

[5] Khalil M.M., Tremoleda J.L., Bayomy T.B., Gsell W. (2011). Molecular SPECT Imaging: An Overview. Int. J. Mol. Imaging.

[6] Chakravarty R., Chakraborty S. (2021). Production of a broad palette of positron emitting radioisotopes using a low-energy cyclotron: Towards a new success story in cancer imaging?. Appl. Radiat. Isot..

[7] Dash A., Chakravarty R. (2019). Radionuclide generators: The prospect of availing PET radiotracers to meet current clinical needs and future research demands. Am. J. Nucl. Med. Mol. Imaging.

[8] Benard F., Buckley K.R., Ruth T.J., Zeisler S.K., Klug J., Hanemaayer V., Vuckovic M., Hou X., Celler A., Appiah J.P. (2014). Implementation of Multi-Curie Production of (99m)Tc by Conventional Medical Cyclotrons. J. Nucl. Med..

[9] Kidane B., Zabel P.L., Gupta V., Whiston C., Wright F., Brackstone M. (2015). Cysteine rhenium colloid: A novel radiocolloid for identifying sentinel lymph nodes in breast cancer surgery. Clin. Breast. Cancer.

[10] Wei L., Bensimon C., Lockwood J., Yan X., Fernando P., Wells R.G., Duan Y., Chen Y.X., Redshaw J.R., Covitz P.A. (2013). Synthesis and characterization of 123I-CMICE-013: A potential SPECT myocardial perfusion imaging agent. Bioorganic Med. Chem..

[11] Lekx K.S., deKemp R.A., Beanlands R.S., Wisenberg G., Wells R.G., Stodilka R.Z., Lortie M., Klein R., Zabel P., Kovacs M.S. (2010). Quantification of regional myocardial blood flow in a canine model of stunned and infarcted myocardium: Comparison of rubidium-82 positron emission tomography with microspheres. Nucl. Med. Commun..

[12] Lekx K.S., de Kemp R.A., Beanlands R.S., Wisenberg G., Wells G., Stodilka R.Z., Lortie M., Klein R., Zabel P., Kovacs M.S. (2010). 3D versus 2D dynamic 82Rb myocardial blood flow imaging in a canine model of stunned and infarcted myocardium. Nucl. Med. Commun..

[13] Williams K.A. (2005). Measurement of ventricular function with scintigraphic techniques: Part I-imaging hardware, radiopharmaceuticals, and first-pass radionuclide angiography. J. Nucl. Cardiol..

[14] Williams K.A. (2005). A historical perspective on measurement of ventricular function with scintigraphic techniques: Part II--Ventricular function with gated techniques for blood pool and perfusion imaging. J. Nucl. Cardiol..

[15] Winzelberg G.G., Castronovo F.P., Callahan R.J., McKusick K.A., Strauss H.W. (1980). ^111^In oxine labeled red cells for detection of simulated lower gastrointestinal bleeding in an animal model. Radiology.

[16] Srivastava S.C., Chervu L.R. (1984). Radionuclide-labeled red blood cells: Current status and future prospects. Semin. Nucl. Med..

[17] Birnbaum B.A., Weinreb J.C., Megibow A.J., Sanger J.J., Lubat E., Kanamuller H., Noz M.E., Bosniak M.A. (1990). Definitive diagnosis of hepatic hemangiomas: MR imaging versus Tc-99m-labeled red blood cell SPECT. Radiology.

[18] Satpati D. (2021). Recent Breakthrough in (68)Ga-Radiopharmaceuticals Cold Kits for Convenient PET Radiopharmacy. Bioconjugate Chem..

[19] Knesaurek K., Machac J., Krynyckyi B.R., Almeida O.D. (2003). Comparison of 2-dimensional and 3-dimensional 82Rb myocardial perfusion PET imaging. J. Nucl. Med..

[20] Nelson B.J.B., Andersson J.D., Wuest F., Spreckelmeyer S. (2022). Good practices for (68)Ga radiopharmaceutical production. EJNMMI Radiopharm. Chem..

[21] Conti M., Eriksson L. (2016). Physics of pure and non-pure positron emitters for PET: A review and a discussion. EJNMMI Phys..

[22] Hambye A.S., Vandermeiren R., Vervaet A., Vandevivere J. (1995). Failure to label red blood cells adequately in daily practice using an in vivo method: Methodological and clinical considerations. Eur. J. Nucl. Med..

[23] Wangler B., Quandt G., Iovkova L., Schirrmacher E., Wangler C., Boening G., Hacker M., Schmoeckel M., Jurkschat K., Bartenstein P. (2009). Kit-like 18F-labeling of proteins: Synthesis of 4-(di-tert-butyl [18F]fluorosilyl)benzenethiol (Si [18F]FA-SH) labeled rat serum albumin for blood pool imaging with PET. Bioconjugate Chem..

[24] Basuli F., Li C., Xu B., Williams M., Wong K., Coble V.L., Vasalatiy O., Seidel J., Green M.V., Griffiths G.L. (2015). Synthesis of fluorine-18 radio-labeled serum albumins for PET blood pool imaging. Nucl. Med. Biol..

[25] Wängler C., Wängler B., Lehner S., Elsner A., Todica A., Bartenstein P., Hacker M., Schirrmacher R. (2011). A universally applicable 68Ga-labeling technique for proteins. J. Nucl. Med..

[26] Feng L., Fang J., Zeng X., Liu H., Zhang J., Huang L., Guo Z., Zhuang R., Zhang X. (2022). (68)Ga-Labeled Maleimide for Blood Pool and Lymph PET Imaging through Covalent Bonding to Serum Albumin In Vivo. ACS Omega.

[27] Zhang J., Lang L., Zhu Z., Li F., Niu G., Chen X. (2015). Clinical Translation of an Albumin-Binding PET Radiotracer 68Ga-NEB. J. Nucl. Med..

[28] Niu G., Lang L., Kiesewetter D.O., Ma Y., Sun Z., Guo N., Guo J., Wu C., Chen X. (2014). In Vivo Labeling of Serum Albumin for PET. J. Nucl. Med..

[29] Frey H., Haag R. (2002). Dendritic polyglycerol: A new versatile biocompatible-material. Rev. Mol. Biotechnol..

[30] Kainthan R.K., Gnanamani M., Ganguli M., Ghosh T., Brooks D.E., Maiti S., Kizhakkedathu J.N. (2006). Blood compatibility of novel water soluble hyperbranched polyglycerol-based multivalent cationic polymers and their interaction with DNA. Biomaterials.

[31] Kainthan R.K., Janzen J., Kizhakkedathu J.N., Devine D.V., Brooks D.E. (2008). Hydrophobically derivatized hyperbranched polyglycerol as a human serum albumin substitute. Biomaterials.

[32] Schmitt V., Rodriguez-Rodriguez C., Hamilton J.L., Shenoi R.A., Schaffer P., Sossi V., Kizhakkedathu J.N., Saatchi K., Häfeli U.O. (2018). Quantitative SPECT Imaging and Biodistribution Point to Molecular Weight Independent Tumor Uptake For Some Long-Circulating Polymer Nanocarriers. RSC Adv..

[33] Kainthan R.K., Muliawan E.B., Hatzikiriakos S.G., Brooks D.E. (2006). Synthesis, characterization, and viscoelastic properties of high molecular weight hyperbranched polyglycerols. Macromolecules.

[34] Saatchi K., Gelder N., Gershkovich P., Sivak O., Wasan K.M., Kainthan R.K., Brooks D.E., Häfeli U.O. (2012). Long-circulating nontoxic cardiac blood pool imaging agent based on hyperbranched polyglycerols. Int. J. Pharm..

[35] Stabin M.G., Sparks R.B., Crowe E. (2005). OLINDA/EXM: The second-generation personal computer software for internal dose assessment in nuclear medicine. J. Nucl. Med..

[36] Siegel J.A., Thomas S.R., Stubbs J.B., Stabin M.G., Hays M.T., Koral K.F., Robertson J.S., Howell R.W., Wessels B.W., Fisher D.R. (1999). MIRD pamphlet no. 16: Techniques for quantitative radiopharmaceutical biodistribution data acquisition and analysis for use in human radiation dose estimates. J. Nucl. Med..

[37] Vanhove C., Lahoutte T., Defrise M., Bossuyt A., Franken P.R. (2005). Reproducibility of left ventricular volume and ejection fraction measurements in rat using pinhole gated SPECT. Eur. J. Nucl. Med. Mol. Imaging.

[38] Croteau E., Benard F., Cadorette J., Gauthier M.E., Aliaga A., Bentourkia M., Lecomte R. (2003). Quantitative gated PET for the assessment of left ventricular function in small animals. J. Nucl. Med..

[39] Stegger L., Heijman E., Schafers K.P., Nicolay K., Schafers M.A., Strijkers G.J. (2009). Quantification of left ventricular volumes and ejection fraction in mice using PET, compared with MRI. J. Nucl. Med..

[40] Hurford W.E., Crosby G., Strauss H.W., Jones R., Lowenstein E. (1990). Ventricular performance and glucose uptake in rats during chronic hypobaric hypoxia. J. Nucl. Med..

[41] Redfors B., Shao Y., Omerovic E. (2014). Influence of anesthetic agent, depth of anesthesia and body temperature on cardiovascular functional parameters in the rat. Lab. Anim..

[42] Cicone F., Viertl D., Quintela Pousa A.M., Denoël T., Gnesin S., Scopinaro F., Vozenin M.-C., Prior J.O. (2017). Cardiac Radionuclide Imaging in Rodents: A Review of Methods, Results, and Factors at Play. Front. Med..

[43] Stabin M.G. (2008). Radiopharmaceuticals for nuclear cardiology: Radiation dosimetry, uncertainties, and risk. J. Nucl. Med..

[44] Corbett R.H. (2008). Ethical issues, justification, referral criteria for budget limited and high-dose procedures. Radiat. Prot. Dosim..

[45] Dam H.Q., Brandon D.C., Grantham V.V., Hilson A.J., Howarth D.M., Maurer A.H., Stabin M.G., Tulchinsky M., Ziessman H.A., Zuckier L.S. (2014). The SNMMI Procedure Standard/EANM Practice Guideline for Gastrointestinal Bleeding Scintigraphy 2.0. J. Nucl. Med. Technol..

[46] Kalender W.A. (2014). Dose in x-ray computed tomography. Phys. Med. Biol..

[47] Cherry S.R., Jones T., Karp J.S., Qi J., Moses W.W., Badawi R.D. (2018). Total-Body PET: Maximizing Sensitivity to Create New Opportunities for Clinical Research and Patient Care. J. Nucl. Med..

[48] Badawi R.D., Shi H., Hu P., Chen S., Xu T., Price P.M., Ding Y., Spencer B.A., Nardo L., Liu W. (2019). First Human Imaging Studies with the EXPLORER Total-Body PET Scanner. J. Nucl. Med..

[49] Thrall J.H., Freitas J.E., Swanson D., Rogers W.L., Clare J.M., Brown M.L., Pitt B. (1978). Clinical comparison of cardiac blood pool visualization with technetium-99m red blood cells labeled in vivo and with technetium-99m human serum albumin. J. Nucl. Med..

[50] Boros E., Ferreira C.L., Cawthray J.F., Price E.W., Patrick B.O., Wester D.W., Adam M.J., Orvig C. (2010). Acyclic chelate with ideal properties for ^68^Ga PET imaging agent elaboration. J. Am. Chem. Soc..

[51] Hoffend J., Mier W., Schuhmacher J., Schmidt K., Dimitrakopoulou-Strauss A., Strauss L.G., Eisenhut M., Kinscherf R., Haberkorn U. (2005). Gallium-68-DOTA-albumin as a PET blood-pool marker: Experimental evaluation in vivo. Nucl. Med. Biol..

[52] Cross S.J., Lee H.S., Metcalfe M.J., Norton M.Y., Evans N.T., Walton S. (1994). Assessment of left ventricular regional wall motion with blood pool tomography: Comparison of 11CO PET with 99Tcm SPECT. Nucl. Med. Commun..

[53] Hofman H.A., Knaapen P., Boellaard R., Bondarenko O., Gotte M.J., van Dockum W.G., Visser C.A., van Rossum A.C., Lammertsma A.A., Visser F.C. (2005). Measurement of left ventricular volumes and function with O-15-labeled carbon monoxide gated positron emission tomography: Comparison with magnetic resonance imaging. J. Nucl. Cardiol..

[54] Cardinal Health Ga-68 Generator. https://www.cardinalhealth.com/en/product-solutions/pharmaceutical-products/nuclear-medicine/radiopharmaceuticals/pet/ga-68.html.

[55] RadioMedix Gallium-68 Generator. https://radiomedix.com/products/gallium-68-generator.

[56] Kozempel J., Mokhodoeva O., Vlk M. (2018). Progress in Targeted Alpha-Particle Therapy. What We Learned about Recoils Release from In Vivo Generators. Molecules.

