# Supplementary material for: Preclinical PET Imaging and Toxicity Study of a 68Ga-Functionalized Polymeric Cardiac Blood Pool Agent
Source: Pharmaceutics. 2023 Feb 25;15(3):767. doi: 10.3390/pharmaceutics15030767 (PMC10052923; doi:10.3390/pharmaceutics15030767)
Supplement: Supplementary file 1 [file pharmaceutics-15-00767-s001.zip › Updated pharmaceutics-2180302-supplementary.pdf]

Supplemental Information

Preclinical PET Imaging and Toxicity Study of a ^{68}Ga-Functionalized Polymeric Cardiac Blood Pool Agent

Katayoun Saatchi, PhD[1]*, François Bénard, MD, PhD[2,3], Navjit Hundal[3], Joshua Grimes[2], Sergey Shcherbinin, PhD[2], Maral Pourghiasian[3], Donald E. Brooks, PhD[4], Anna Celler, PhD[2], and Urs O. Häfeli, PhD[1]*

[1]Faculty of Pharmaceutical Sciences, University of British Columbia, Vancouver, BC V6T 1Z3, Canada, [2]Department of Radiology, University of British Columbia, Vancouver, BC V5Z 1M9, Canada, [3]BC Cancer, Vancouver, BC V5Z 4E6, Canada, [4]Department of Pathology and Laboratory Medicine, University of British Columbia, Vancouver, BC V6T 1Z3 Canada

Keywords: Ga-68, hyperbranched polyglycerol, non-toxic polymer, macromolecule, blood pool imaging agent, PET radiopharmaceutical, internal dosimetry

* Corresponding authors: urs.hafeli@ubc.ca (U.O.H.); kathy.saatchi@ubc.ca (K.S.)

Supplemental Video S1:

The attached video shows a representative movie of the beating heart of a healthy Sprague Dawley rat reconstructed from the gated ^{68}Ga-HPG PET images.

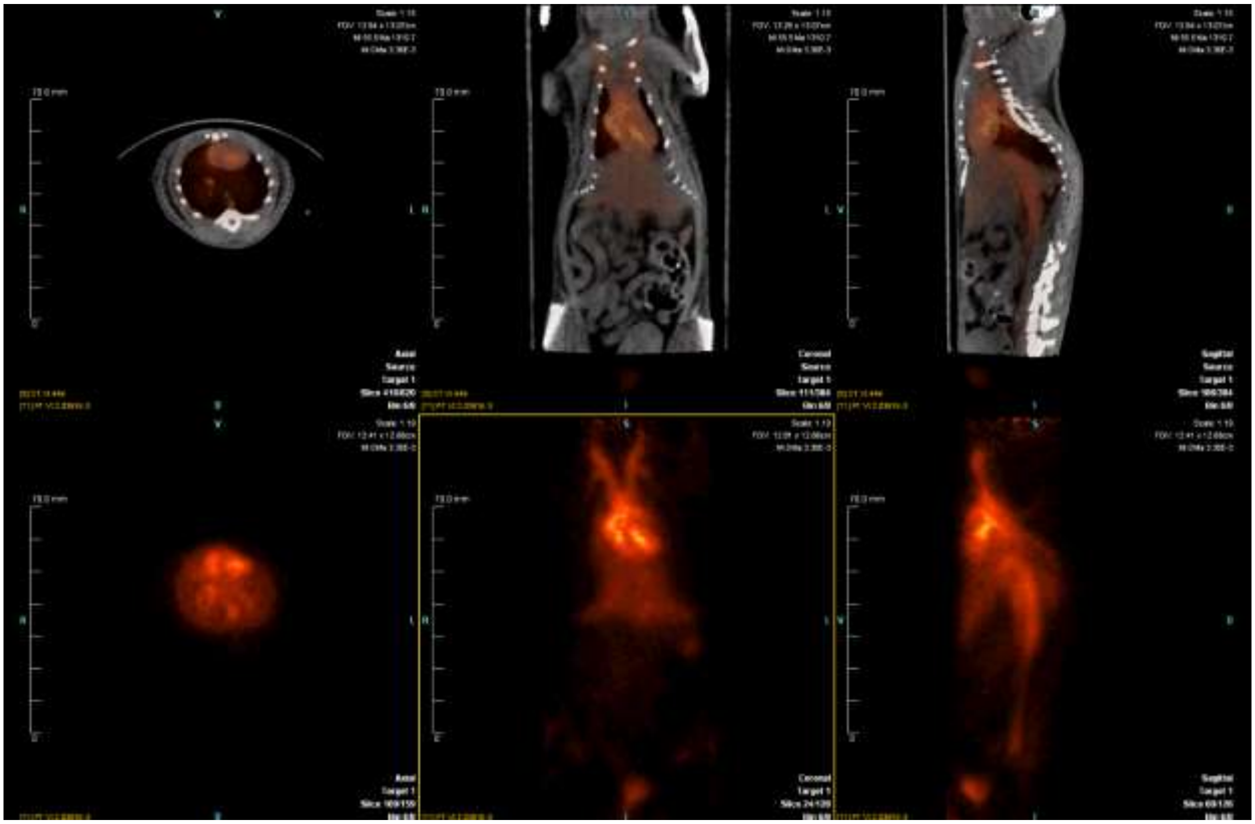

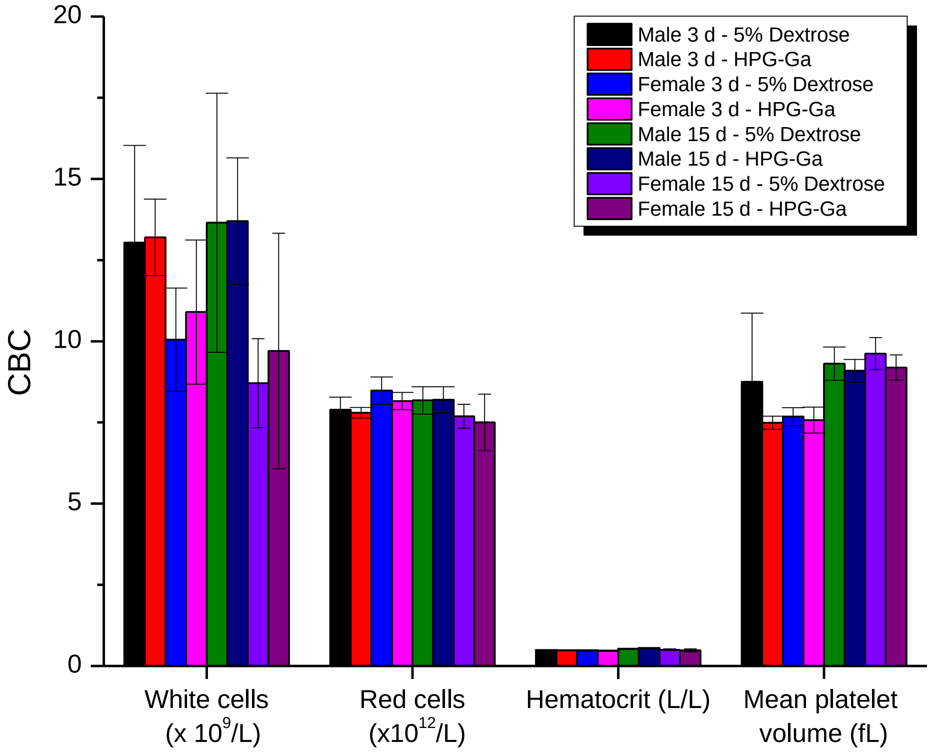

Figure S1.

Analysis of complete blood counts (CBC) on Day 3 and 15 from all Sprague Dawley rats (n = 10 per group). The values shown here include the number of white and red cells, the hematocrit and the mean platelet volume.

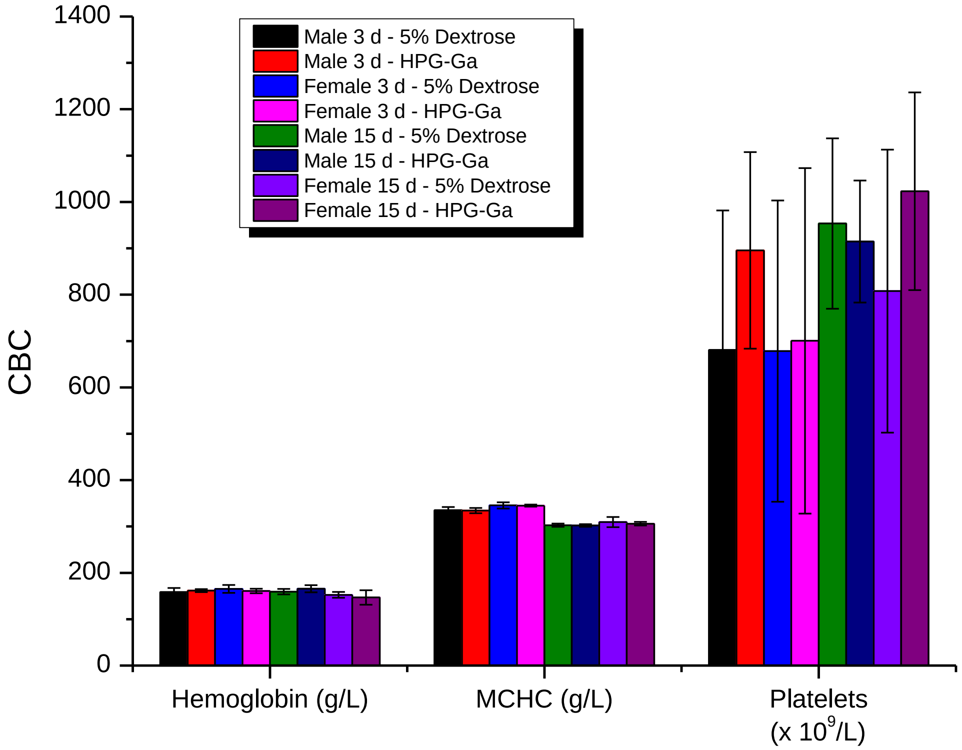

Figure S2.

Additional values determined during the CBC including the hemoglobin concentration, the mean corpuscular hemoglobin concentration (MCHC) and the platelets number.

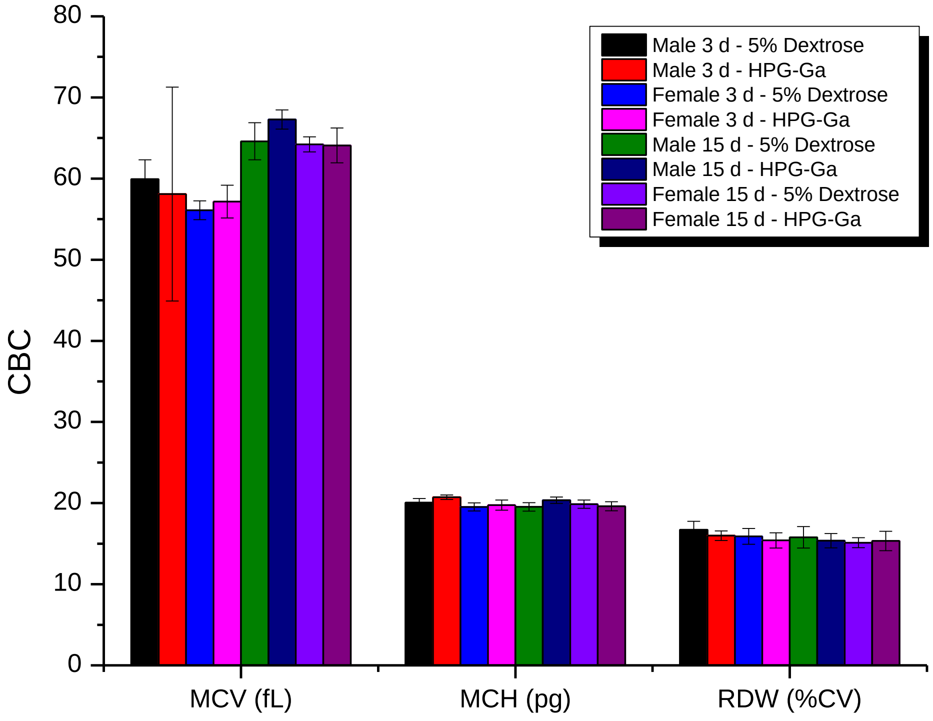

Figure S3.
Additional values determined during the CBC including the mean corpuscular volume (MCV), mean corpuscular hemoglobin (MCH) and the red cell distribution width (RDW).
